# Fast NJ-like algorithms to deal with incomplete distance matrices

**DOI:** 10.1186/1471-2105-9-166

**Published:** 2008-03-26

**Authors:** Alexis Criscuolo, Olivier Gascuel

**Affiliations:** 1 Equipe Méthodes et Algorithmes pour la Bioinformatique, LIRMM, CNRS – Université Montpellier 2, 161 rue Ada, 34392 Montpellier Cedex 05, France; 2 Groupe Phylogénie Moléculaire, ISEM, CNRS – Université Montpellier 2, C.C. 064, 34095 Montpellier Cedex 05, France; 3 Equipe Bioinformatique Théorique, LSIIT, Université Louis Pasteur, Strasbourg 1, Pôle API, Boulevard Sébastien Brant, BP 10413, 67412 Illkirch Cedex, France

## Abstract

**Background:**

Distance-based phylogeny inference methods first estimate evolutionary distances between every pair of taxa, then build a tree from the so-obtained distance matrix. These methods are fast and fairly accurate. However, they hardly deal with incomplete distance matrices. Such matrices are frequent with recent multi-gene studies, when two species do not share any gene in analyzed data. The few existing algorithms to infer trees with satisfying accuracy from incomplete distance matrices have time complexity in *O*(*n*^4^) or more, where *n *is the number of taxa, which precludes large scale studies. Agglomerative distance algorithms (e.g. NJ [1,2]) are much faster, with time complexity in *O*(*n*^3^) which allows huge datasets and heavy bootstrap analyses to be dealt with. These algorithms proceed in three steps: (a) search for the taxon pair to be agglomerated, (b) estimate the lengths of the two so-created branches, (c) reduce the distance matrix and return to (a) until the tree is fully resolved. But available agglomerative algorithms cannot deal with incomplete matrices.

**Results:**

We propose an adaptation to incomplete matrices of three agglomerative algorithms, namely NJ, BIONJ [3] and MVR [4]. Our adaptation generalizes to incomplete matrices the taxon pair selection criterion of NJ (also used by BIONJ and MVR), and combines this generalized criterion with that of ADDTREE [5]. Steps (b) and (c) are also modified, but *O*(*n*^3^) time complexity is kept. The performance of these new algorithms is studied with large scale simulations, which mimic multi-gene phylogenomic datasets. Our new algorithms – named NJ*, BIONJ* and MVR* – infer phylogenetic trees that are as least as accurate as those inferred by other available methods, but with much faster running times. MVR* presents the best overall performance. This algorithm accounts for the variance of the pairwise evolutionary distance estimates, and is well suited for multi-gene studies where some distances are accurately estimated using numerous genes, whereas others are poorly estimated (or not estimated) due to the low number (absence) of sequenced genes being shared by both species.

**Conclusion:**

Our distance-based agglomerative algorithms NJ*, BIONJ* and MVR* are fast and accurate, and should be quite useful for large scale phylogenomic studies. When combined with the SDM method [6] to estimate a distance matrix from multiple genes, they offer a relevant alternative to usual supertree techniques [7]. Binaries and all simulated data are downloadable from [8].

## Background

Phylogeny inference methods can be classified into two main categories: character-based (e.g. maximum-parsimony or maximum-likelihood) and distance-based approaches. The latter have low running times which are quite useful (mandatory in some cases) to perform large-scale studies and bootstrap analyses. A number of computer simulations [9-17] have shown that distance methods are fairly accurate, though not as accurate as likelihood-based methods that are much more time consuming. Using any distance-based method first requires to estimate the pairwise evolutionary distances between every taxon pair. These distances are usually estimated from DNA, RNA or protein sequences, but can also be obtained from DNA-DNA hybridization experiments or, e.g., computed from morphological data (see [18] for a review on distance estimation from various data types).

In the last few years, phylogenomic studies (i.e. phylogeny reconstruction from large gene collections [7]) have instigated to the development of fast tree-building techniques being able to infer trees from datasets comprising hundreds of genes and taxa. The *low-level gene combination *involves concatenating the different genes into a unique *supermatrix of characters*, and then analyzing this matrix with a standard tree building method. This approach was shown to perform poorly when combined with distance methods, due to inaccurate distance estimations from such large heterogeneous character matrix [6]. Better distance-based trees are obtained by extracting the phylogenetic information from each gene separately, and then combining resulting information sources into a unique *distance supermatrix*. The *Average Consensus Supertree *(ACS [19]) and *Super Distance Matrix *(SDM [6]) techniques input a collection of distance matrices being estimated from each gene separately (the so-called *medium-level combination*), or being equivalent to the gene trees (the *high-level combination*). These distance matrices are deformed, without modifying their topological message, and then averaged to obtain the distance supermatrix, which is finally analyzed using a distance-based tree building algorithm.

Estimating the distance supermatrix is fast. However, missing entries may occur in distance supermatrices depending on the extent of taxon overlap within the source matrices. For example, with the two large data sets of Driskell et al. [20], which were collected from Swiss-Prot and Gen-Bank thanks to a computer program, the ratio of missing distances is ~19% and ~1.2%, respectively. These distances are missing because only a few genes are sequenced within each species, meaning that a number of species pairs do not share any sequenced gene in common and cannot be compared using available data. However, Driskell et al. showed that, despite the sparseness of data and the fact that only a small subset of these data is potentially phylogenetically informative, a topological signal still emerges, which provides useful insights into the tree of life (see [20] and below for details). Analogous findings were reported by a number of authors in various contexts [21-23], and tree building from sparse data has become topical, as can be seen from the flourishing literature on supertrees.

However, tree building from incomplete distance matrices is NP-hard [24], and thus practical algorithms are heuristics. The indirect approach involves first estimating missing distances by applying an ultrametric [25], additive [26], decomposition-based [27], or quartet-based [28] completion algorithm. The TREX package [29] provides several implementations of such algorithms to be used before tree building using any standard method with the completed matrix. The direct approach involves using a weighted least-squares (WLS) algorithm and associating missing distances with null weight (i.e. infinite variance), which means that missing distances are simply discarded from WLS computations ([18], pp. 449). The FITCH algorithm [30] from the PHYLIP package [31] and the MWMODIF algorithm [32] from TREX implement this technique. A combination of both direct and indirect methods is provided by MW* [33] (also available in TREX); this algorithm first applies an ultrametric or additive completion algorithm (depending on the density of missing distances) and then infers a tree using MWMODIF, where weights are set to 1.0 for known distances, 0.5 for estimated distances, and 0.0 for missing distances (if any remain). All these (direct or indirect) algorithms have *O*(*n*^4^) time complexity or more, where *n *is the number of taxa. This limits their application to medium-sized datasets (say 200 taxa without bootstrapping, see below).

Agglomerative algorithms are much faster and allow dealing with thousands of taxa, as soon as the distance matrix is complete. The most popular of them is the Neighbor-Joining (NJ) algorithm [1,2]. Starting from a star tree, agglomerative algorithms iteratively perform the three following steps, until the tree is completely resolved:

(a) select a taxon pair *xy *that is agglomerated into a new node *u*;

(b) estimate the length of the two so-created external branches *ux *and *uy*;

(c) replace *x *and *y *by *u *in the distance matrix, and estimate the new distances between *u *and the not-yet-agglomerated taxa.

Step (a) is more time consuming than the two other steps, because of testing all the *O*(*n*^2^) taxon pairs to select the optimal one. To this purpose, NJ optimizes a numerical criterion that is denoted as *Q*_*xy*_. This criterion admits several interpretations related to the Minimum Evolution principle [1,34], but also to the acentrality of the considered pair [35,36]. In this last interpretation (used here), *Q*_*xy *_measures how much the path joining *x *to *y *is far from the other taxa *i *≠ *x*, *y*. The *xy *pair maximizing *Q*_*xy *_corresponds to the two taxa which are most distant from the other ones and is the best candidate for agglomeration. Another criterion, denoted as *N*_*xy*_, is used by ADDTREE [5]; this second criterion is based on the four point condition [37,38] and counts the number of taxon quartets *xyij *where *x *and *y *are neighbors. When the distance matrix exactly corresponds to a tree (it is then said to be *additive*), *N*_*xy *_indicates all pairs of sibling taxa in the tree, whereas *Q*_*xy *_indicates just one such taxon pair. We shall see that this property of *N*_*xy *_is a great advantage when dealing with incomplete distance matrices. Indeed, *Q*_*xy *_is sometimes unusable whereas *N*_*xy *_is still informative.

Steps (b) and (c) essentially correspond to distance averaging, which requires *O*(*n*) run time. These three steps being repeated *n *- 2 times, agglomerative algorithms require *O*(*n*^3^) time when using the *Q*_*xy *_pair selection criterion, and *O*(*n*^4^) with *N*_*xy *_[39].

Several refinements of the NJ algorithm have been proposed. BIONJ [3] minimizes the variances associated to the new distances being estimated during each reduction step (c). This way, BIONJ makes use at each iteration of reliable distance estimates to select the new taxon pairs to be agglomerated. To this aim, BIONJ uses a simple Poisson model of the variances and covariances of the distances being contained in the initial distance matrix. BIONJ was generalized into the *Minimum Variance Reduction *algorithm (MVR [4]), a WLS variant of which can deal with any distance variance model, but which does not account for the distance covariances. It has been shown using computer simulations that this variant (named WLS-MVR in [4] but referred here as MVR for simplicity) has similar accuracy as NJ when applied to distance matrices estimated from one-gene alignments [4]. WEIGHBOR [40] further refines BIONJ approach and uses an agglomeration criterion which accounts for the variances of evolutionary distances. All these algorithms require *O*(*n*^3^) time. Faster, sophisticated distance-based algorithms have been proposed in the last few years [41-46], some of them being clearly more accurate than NJ and BIONJ (e.g. FASTME [42] and STC [44], in *O*(*n*^2 ^log(*n*)) and *O*(*n*^2^), respectively).

In this paper, we propose an adaptation of the agglomerative scheme to quickly infer phylogenetic trees from incomplete distance matrices. We show that the *Q*_*xy *_criterion may be rewritten to express the mean acentrality of the *xy *taxon pair. In the same way, the *N*_*xy *_criterion may be rewritten to express the mean number of taxon quartets where *x *and *y *are neighbors. By estimating these two means using all available (non-missing) distances, we define the two criteria $Q_{xy}^{*}$ and $N_{xy}^{*}$ which allow for the selection of taxon pairs in step (a), even when the distance matrix is incomplete. Using these two new criteria in the agglomerative scheme requires *O*(*n*^3^) and *O*(*n*^4^) run time, respectively. A limitation of $Q_{xy}^{*}$ and $N_{xy}^{*}$ is that they cannot be computed when the distance corresponding to the *xy *pair is missing (see Methods for more). However, this difficulty is inherent to the problem of building trees from incomplete distance matrices and is encountered (in various forms) by all methods to deal with this problem. Moreover, $N_{xy}^{*}$ partly circumvents this difficulty thanks to its ability to indicate several relevant pairs, rather than a single one with $Q_{xy}^{*}$ (see Methods for more). As running $N_{xy}^{*}$ requires *O*(*n*^4^) time, we use a filtering technique: at each step (a) we use $Q_{xy}^{*}$ to select the *s *most promising pairs for agglomeration, and then use $N_{xy}^{*}$ to select the best of these *s *pairs. This computational trick (and other refinements, see Methods) greatly improves the accuracy compared to using $Q_{xy}^{*}$ only, while requiring *O*(*sn*^3^) time, where *s *is a small constant (*s *= 15 in our experiments). Finally, the original NJ, BIONJ and MVR formulae corresponding to steps (b) and (c) essentially are distance averaging and are easily adapted to incomplete matrices. The three new algorithms are named NJ*, BIONJ* and MVR*, respectively.

## Results and Discussion

Several computer simulations are presented in this section to assess the performance of NJ*, BIONJ* and MVR*. We first compare the agglomeration criteria $Q_{xy}^{*}$, $N_{xy}^{*}$ and their combination with distance matrices that are additive, but contain missing entries. Then, using more realistic datasets, we compare NJ*, BIONJ*, MVR* to FITCH [30] and MW* [33], in terms of both topological accuracy and run times.

### Comparison of agglomeration criteria

Our approach is similar to Makarenkov and Lapointe's [33]. We analyze with various algorithms and criteria a distance matrix with randomly deleted entries. The distance matrix we use is additive, i.e. is obtained from a tree by computing the path length distance between every taxon pair. Let *T *denote this tree and (*T*_*ij*_) be the corresponding distance matrix, where *T*_*ij *_is the path-length (or patristic) distance between taxa *i *and *j *in *T*. When no entry is missing, such an additive matrix uniquely defines *T*, which is recovered by any consistent algorithms (as are all algorithms being tested here). When entries are missing in (*T*_*ij*_), recovering *T *becomes a difficult task (see above), and we measure how well the algorithms perform when given an increasing number of missing distances. Such data thus are not realistic from a biological stand point, as evolutionary distances estimated from sequences are not additive, but this is a simple and standard approach to compare algorithms and agglomeration criteria.

We use for the correct tree *T *the phylogeny of 75 placental mammals from [6]. The percentage of missing entries is *P*_miss _= 1%, 5%, 10%, 20%, 30%. For each *P*_miss _value, 500 replicates are randomly generated. From each of these 5 × 500 incomplete additive distance matrices, a tree $\hat{T}$ is inferred by FITCH, MW* and BIONJ*. Various values of the *s *parameter are tested for BIONJ*, in order to compare the topological accuracy of $Q_{xy}^{*}$, $N_{xy}^{*}$, and of the combination of these two agglomeration criteria. With *s *= 1, BIONJ* uses $Q_{xy}^{*}$ only. With, *s *> 1, the taxon pairs corresponding to the *s *highest values of $Q_{xy}^{*}$ are reanalyzed with $N_{xy}^{*}$ (and with other criteria when ties occur; see Methods). When *s *becomes large (which is denoted as *s *= max) BIONJ* uses $N_{xy}^{*}$ only, as all taxon pairs are retained in the first selection step.

**Figure 1:**
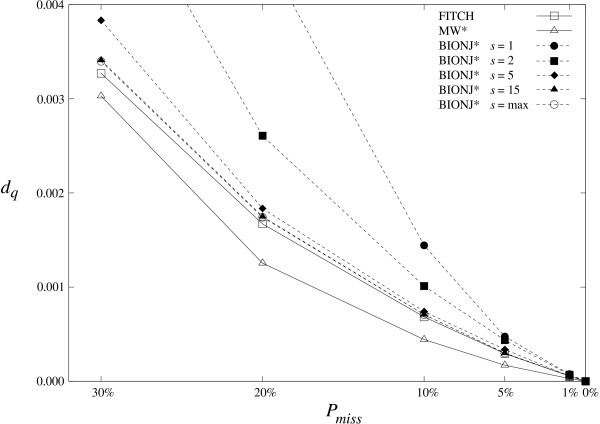
**Topological accuracy depending on the rate of missing entries**. Horizontal axis: percentage of missing distances (*P*_miss_. Vertical axis: topological accuracy measured by the mean (over 500 trials) quartet distance (*d*_*q*_) between the correct and inferred trees. *s*: number of taxon pairs that BIONJ* first selects using NJ-like $Q_{xy}^{*}$ criterion (6), and then analyzes using score-based $N_{xy}^{*}$ criterion (9) (and criteria (8), (10), (11) in case of ties). The distance matrix is additive, and thus all methods recover the correct tree when *P*_miss _= 0.

Each inferred tree $\hat{T}$ is compared to the correct tree *T *by using the quartet distance *d*_*q *_[47]. This topological distance measures the number of resolved 4-taxon subtrees which are induced by one tree but not the other, and thus is more precise than the widely used bipartition distance [48] which counts the number of internal branches present in one tree but not in the other. Moreover, the quartet distance is less affected than the bipartition distance by small topological errors, e.g. wrong position of a single taxon [49]. This distance is normalized: *d*_*q *_= 0 indicates that *T *and $\hat{T}$ are identical, whereas *d*_*q *_= 1 means that both trees do not share any resolved 4-taxon subtrees. Averages of the 500 *d*_*q *_measures for each *P*_miss _value are displayed in Figure 1, for FITCH, MW*, and BIONJ* with various *s *values.

All curves in Figure 1 are decreasing; as expected, the correct tree *T *is better recovered (i.e. the mean *d*_*q *_value between $\hat{T}$ and *T *decreases) as the proportion of missing distance *P*_*miss *_becomes closer to 0. Using $N_{xy}^{*}$ in BIONJ* greatly improves the agglomeration step; e.g. with *P*_miss _= 10%, mean *d*_*q *_values of BIONJ* are ~0.0015 and ~0.0008, with *s *= 1 and *s *= 15, respectively. However, there is no significant difference between *s *= 15 and *s *= max (as assessed by a sign-test [50] based on the 500 replicates, all *p*-values are much larger than 0.05), meaning that a small value of *s *(e.g. *s *= 15) seems to be enough to focus on the most relevant pairs, while avoiding the computational burden of using $N_{xy}^{*}$ only. Further experiments (see below) confirm this finding. FITCH and BIONJ* (with *s *= 15 and *s *= max) have similar accuracy, while MW* tends to perform better than the other algorithms with these data. However, we shall see that algorithm ordering is different with more realistic simulations. These experiments thus confirm the advantage of combining $Q_{xy}^{*}$ and $N_{xy}^{*}$ within BIONJ*, and similar results (not shown) are obtained with NJ* and MVR*.

### Comparison of reconstruction algorithms with distance supermatrices

We re-use a simulation protocol that we have used previously to compare a number of tree-reconstruction methods in a phylogenomic context [6]. This protocol involves generating sequences and evolving them along trees, and is more realistic than the comparison described above. We first summarize this protocol, and then report the results that are obtained with the simulated datasets by FITCH, MW*, NJ*, BIONJ* and MVR*. To estimate the distance supermatrix that is the input of these algorithms, we use the SDM method ([6], see also Methods) which computes a supermatrix that summarizes the topological signal being contained in a collection $\left\{\left(\Delta_{ij}^{1}\right),\left(\Delta_{ij}^{2}\right),...,\left(\Delta_{ij}^{k}\right)\right\}$ of *k *distance matrices. Simulations [6] have shown the high-quality of this distance supermatrix in both medium- and high-level gene combinations.

Simulations are as follows (see [6] for more details). Starting from a randomly generated tree *T *with *n *= 48 taxa, evolution of *k *genes is simulated, with *k *= 2, 4, ..., 20. For each of the *k *genes, some taxa are randomly deleted. Two deletion probabilities are used: 25% to preserve high overlap between the different taxon sets, and 75% to induce low overlap. From these *k *partially deleted gene alignments, *k *distance matrices are estimated to compose the collection *C*_Δ _of source matrices. The SDM method is then run with *C*_Δ _to obtain a distance supermatrix corresponding to a medium-level combination of the *k *partially deleted genes. To study the high-level combination, a phylogenetic tree is inferred by PhyML [17] from each of the *k *partially deleted genes; then, the path length distance between each taxon pair for each of the *k *phylogenies is computed, to form the collection *C*_*T *_of *k *additive distance matrices that are equivalent to the *k *PhyML trees. Finally, SDM is applied to *C*_*T *_to obtain a distance supermatrix corresponding to a high-level gene combination.

This simulation protocol is repeated 500 times for each value of *k *and each deletion proportion. We obtain this way (10 gene collection sizes × 500 collections × 2 overlap conditions × 2 gene combination levels) = 20,000 distance supermatrices, which are denoted as ($\Delta_{ij}^{\text{SDM}}$) and are frequently incomplete. Indeed, if taxon *i *is missing for gene *p*, then $\Delta_{ij}^{p}$ is missing – which is denoted as $\Delta_{ij}^{p}$ = ∅—, and if $\Delta_{ij}^{p}$ = ∅ for all *p *= 1,2, ..., *k*, then $\Delta_{ij}^{\text{SDM}}$ = ∅. With 25% deletion rate, almost all distance supermatrices are complete when *k *≥ 14. With 75% deletion rate, all distance supermatrices are incomplete, but the number of missing distances decreases as *k *increases (missing distance proportions range from 42% to 11%).

FITCH and MW* are run with default options. In accordance with Figure 1, *s *is set to 15 for NJ*, BIONJ* and MVR*. With BIONJ*, *V*_*ij *_variances (associated with $\Delta_{ij}^{\text{SDM}}$ distance estimates) are naturally defined by *V*_*ij *_∝ $\Delta_{ij}^{\text{SDM}}$ if $\Delta_{ij}^{\text{SDM}}$ ≠ ∅, else *V*_*ij *_= ∅. Variances used by MVR* comply with the same rule, but account for other parameters such as the length and the number of sequences being used to estimate each $\Delta_{ij}^{\text{SDM}}$ distance (see Methods). Accuracy of the five algorithms is measured with the topological distance *d*_*q*_, as above, and averaged for the 500 replicates corresponding to each of the conditions. Results are reported in Table 1 for the medium-level gene combination, and in Table 2 for the high-level gene combination. For each value of *k*, the first- and second-best mean *d*_*q *_values are indicated in bold&underlined and bold, respectively, and a sign-test [50] based on the 500 replicates is used to assess the significance of the difference between these two best values.

**Table 1:** Topological accuracy with medium-level distance supermatrices

**(a): 25% taxon deletion rate**						
***k ***=	**FITCH**	MW*	NJ*	BIONJ*	MVR*	***p*-value**

2	**0.0841**	0.0906	0.0926	**0.0841**	0.0857	0.286
4	**0.0504**	0.0546	0.0595	**0.0494**	0.0524	0.466
6	**0.0400**	0.0445	0.0454	**0.0370**	0.0410	0.015
8	0.0330	0.0356	0.0386	**0.0318**	**0.0320**	0.958
10	**0.0271**	0.0300	0.0317	**0.0265**	0.0286	0.364
12	**0.0294**	0.0317	0.0354	**0.0284**	0.0314	0.030
14	**0.0245**	0.0266	0.0286	**0.0235**	0.0251	0.816
16	**0.0290**	0.0318	0.0327	**0.0282**	0.0303	0.028
18	**0.0252**	0.0278	0.0280	**0.0234**	0.0265	0.020
20	**0.0242**	0.0259	0.0281	**0.0230**	0.0247	0.955

						

**(b): 75% taxon deletion rate**						

***k ***=	**FITCH**	MW*	NJ*	BIONJ*	MVR*	***p*-value**

2	**0.2154**	0.2174	0.2187	**0.2131**	0.2163	0.920
4	**0.1683**	0.1778	0.1818	**0.1713**	0.1713	0.060
6	**0.1347**	0.1443	0.1534	0.1418	**0.1400**	≈ 0.0
8	**0.1089**	0.1253	0.1302	0.1137	**0.1114**	0.176
10	**0.0878**	0.1039	0.1117	0.0959	**0.0901**	0.033
12	**0.0825**	0.0968	0.1021	0.0875	**0.0842**	0.470
14	**0.0652**	0.0749	0.0850	0.0710	**0.0676**	0.464
16	**0.0583**	0.0731	0.0802	0.0658	**0.0625**	0.335
18	**0.0516**	0.0617	0.0687	**0.0552**	0.0555	0.074
20	**0.0503**	0.0600	0.0682	0.0560	**0.0509**	0.189

In the medium-level combination, distance matrices are directly estimated from each of the genes and then combined (using SDM) into the distance supermatrix. Topolological accuracy is mesured by the mean (over 500 trials) quartet distance (*d*_*q*_) between the correct and inferred trees. *k*: number of genes. *p*-value: sign-test significance when comparing the 500 *d*_*q *_values of the two best methods that are indicated in bold and underlined (1^st ^method) and bold (2^nd ^one)

**Table 2:** Topological accuracy with high-level distance supermatrices

**(a): 25% taxon deletion rate**						
***k ***=	**FITCH**	MW*	NJ*	BIONJ*	MVR*	***p*-value**

2	**0.0558**	0.0561	0.0586	0.0566	**0.0522**	≈ 0.0
4	**0.0337**	0.0345	0.0361	0.0351	**0.0319**	≈ 0.0
6	**0.0253**	0.0265	0.0272	0.0261	**0.0235**	≈ 0.0
8	0.0227	0.0228	**0.0213**	0.0217	**0.0212**	0.094
10	**0.0187**	0.0188	0.0194	0.0192	**0.0171**	0.047
12	**0.0197**	0.0207	0.0215	0.0199	**0.0191**	0.949
14	**0.0160**	0.0164	0.0164	0.0165	**0.0162**	0.882
16	0.0208	**0.0204**	0.0210	0.0213	**0.0206**	≈ 0.0
18	**0.0170**	0.0177	0.0177	**0.0173**	0.0174	0.271
20	0.0162	0.0168	0.0171	**0.0160**	**0.0158**	0.648

						

**(b): 75% taxon deletion rate**						

***k ***=	**FITCH**	MW*	NJ*	BIONJ*	MVR*	***p*-value**

2	0.1876	0.1877	0.1824	**0.1822**	**0.1817**	0.282
4	0.1396	0.1397	0.1390	**0.1381**	**0.1345**	0.018
6	**0.1095**	0.1125	0.1134	0.1119	**0.1065**	0.166
8	**0.0865**	0.0892	0.0926	0.0870	**0.0823**	0.005
10	**0.0690**	0.0739	0.0766	0.0723	**0.0671**	0.023
12	**0.0641**	0.0670	0.0705	0.0677	**0.0616**	0.015
14	**0.0508**	0.0538	0.0567	0.0534	**0.0493**	≈ 0.0
16	**0.0504**	0.0518	0.0554	0.0512	**0.0457**	≈ 0.0
18	**0.0409**	0.0416	0.0485	0.0424	**0.0402**	0.922
20	**0.0403**	0.0435	0.0453	0.0431	**0.0371**	≈ 0.0

In the high-level combination, ML trees are first inferred separately for every genes, and then these trees are turned into path-length distance matrices which are combined (using SDM) into the distance supermatrix. For symbols and notation, see note to Table 1.

In the medium-level gene combination, NJ* and MW* are outperformed by other algorithms. With a 25% deletion rate, BIONJ* has best topological accuracy, followed by FITCH. However, the sign-test indicates that the difference between these two algorithms is moderately significant as the *p*-value is lower than 0.05 for only five *k *values (= 6, 8, 12, 16, and 18). With a 75% deletion rate, FITCH is best, but again the sign-test shows that FITCH, BIONJ* and MVR* are broadly equivalent.

With high-level combination distance supermatrices, NJ* and MW* still tend to be outperformed by other algorithms. BIONJ* is in between, and the best mean *d*_*q*_ values are observed with MVR* which is followed by FITCH. The sign-test broadly confirms the significance of this observation, though the accuracy difference between MVR* and FITCH is relatively low.

Altogether, these experiments show that MVR* is at least as accurate as FITCH, that BIONJ* has similar performance, while NJ* and MW* are behind these three algorithms. Comparing these findings with the results from (see Figure 2 in [6]), we see that (in the high-level framework, Table 2) MVR* is more accurate than the standard Matrix Representation with Parsimony method (MRP, [51,52]), in most cases; e.g. with *k *= 10, MVR* has mean *d*_*q *_values of 0.0171 and 0.0663, for 25% and 75% deletion rate, respectively, while mean *d*_*q *_values of MRP equal 0.0175 and 0.1152. MVR* (combined with SDM) outperforms MRP with sparse information (75% deletion rate and/or low number of genes), while both approaches are nearly equivalent when the information is abundant (25% deletion rate). An explanation [53] of this finding could be that the distance approach not only uses the topology of the source trees (as MRP) but also their branch lengths. Distance-based supertrees thus contain more information than MRP supertrees, which makes a noticeable difference when the information is sparse, but does not impact much the results with abundant information (see also following simulation results).

### Results with simulations based on Driskell et al. [20] dataset

This section aims to measure the accuracy of the different tree building algorithms when applied to simulated datasets being more realistic than those commonly used in a phylogenomic perspective. Most notably, uniformly random gene deletion (used in previous section, following [54]) is not fully realistic because some genes (e.g. cytochrome b) are sequenced for most species, while some other genes are rarely sequenced (or rare among living species). It follows that the gene presence/absence pattern is different with real datasets to this being induced by uniformly random gene deletion (see [20,55-57] for illustrative examples). To this purpose, we use the character supermatrix from Driskell et al. [20], which comprises 69 green plant species and 254 genes, and was built via an automated exploration process of GenBank. This matrix contains a total number of 2777 sequences and has 87% missing characters, which are unequally distributed among taxa. Only 3 taxa have more than 50% genes, whereas 42 have 10% genes or less. In the same way, a few genes are present in most taxa (e.g., the 2 most sequenced genes belong to 59 taxa), whereas other genes are rare (e.g. 121 genes are present in at most 5 taxa). However, these *k *= 254 genes are complementary and the SDM distance supermatrix only contains ~1.2% missing entries. This low proportion of missing entries is favorable to tree reconstruction, but still requires an algorithm able to deal with incomplete matrices.

We use a simulation protocol analogous to that described above [6]. The only difference is the deletion procedure, with random deletion replaced by the gene presence/absence pattern of (see Figure 2B in [20]). We generate 100 datasets this way with *n *= 69 taxa and *k *= 254 genes. From these 100 datasets, we infer 100 distance matrix collections *C*_Δ _and 100 tree collections *C*_*T*_. Each of these 2 × 100 collections is dealt with by SDM, to obtain a distance supermatrix ($\Delta_{ij}^{\text{SDM}}$) that contains the same missing entries as those induced by the original dataset [20]. We use these matrices to compare FITCH, MW*, NJ*, BIONJ* and MVR*, based on *d*_*q *_quartet distance between the correct and inferred trees (see above). Our three algorithms are run with both *s *= 15 and *s *= max. Results of MRP are also computed, using TNT [58] to infer the most parsimonious trees. TNT is run with 25 random addition sequences, TBR branch swapping and ratchet. The MRP supertree is defined in the standard way [59] as the strict consensus of the most parsimonious trees. Results are displayed in Table 3, which is similar to Tables 1 and 2; the first- and second-best mean *d_q_* values are indicated in bold&underlined and bold, respectively, and sign-tests are used to assess the significance of the differences between MVR* (our best algorithm), FITCH and MRP.

**Table 3:** Topological accuracy with datasets generated from Driskell et al. [20]

**(a): medium level**							
**FITCH**	MW*	NJ*	BIONJ*	MVR*		***p*-value**	
						MVR* – FITCH	MVR* – BIONJ*

0.0234	0.0268	0.0289	**0.0227**	**0.0171**		≈ 0.0	≈ 0.0

							

**(b): high level**							

**FITCH**	MW*	NJ*	BIONJ*	MVR*	**MRP**	***p*-value**	
						MVR* – FITCH	MVR* – MRP

0.0161	0.0165	0.0182	0.0172	**0.0101**	**0.0119**	0.001	0.193

(a): Medium-level combination of the distance matrices being directly estimated from the gene sequences. (b): High-level combination; ML trees are first inferred separately for every genes; MRP turns the gene trees into a matrix of partial binary characters, which is analyzed with parsimony; with the other (distance) methods, the gene trees are turned into path-length distance matrices which are combined into the distance supermatrix. All combinations of source distance matrices are achieved using SDM. *p*-value: sign-test significance when comparing the 100 *d*_*q *_values of MVR* (our best algorithm) to those of FITCH and MRP. For other symbols and notation, see note to Table 1.

NJ*, BIONJ* and MVR* do not show any significant difference when used with *s *= 15 and *s *= max (as assessed by the sign-test, all *p*-values are much larger than 0.05, results not shown). This confirms the results of the previous experiments to compare our various agglomeration criteria. NJ* has the worst accuracy, especially in the high-level combination framework. MW*, FITCH and BIONJ* show similar performance, while MVR* is best among distance approaches in the two gene combination levels. Moreover, the difference between MVR* and FITCH is highly significant (sign-test *p*-value ≈ 0.0). In the high-level framework, MVR* tends to be better than MRP, although the information is quite abundant (254 genes, ~1.2% of missing distances); however, the difference is not significant with 100 replicates (sign-test *p*-value ≈ 0.2). The results among distance methods are explained by the fact that MVR* uses fairly accurate estimates ($V_{ij}^{\text{SDM}}$) of the variances of the distances in ($\Delta_{ij}^{\text{SDM}}$). Indeed, dataset [20] induces a highly heterogeneous distribution of missing sequences, meaning that some distances are well estimated thanks to a large number of sequences, while some others are poorly estimated via a few sequences. This is accounted for by MVR* in ($V_{ij}^{\text{SDM}}$) calculations (see Methods), while MW*, FITCH and BIONJ* lack this information and use inaccurate estimations of ($V_{ij}^{\text{SDM}}$). The difference between these two approaches (i.e. MVR* on the one hand, and MW*, FITCH and BIONJ* on the other hand) is somewhat hidden when using uniformly random sequence deletion, because with the latter all distances are broadly estimated with the same number of genes. With biologically realistic pattern of gene presence/absence, the difference becomes important, especially for the high-level combination. Thus, this last set of simulations confirms the findings of the previous ones and supports the capacity of MVR* for dealing with phylogenomic data.

### Run time comparison

Run times with various dataset sizes have been measured on a PC Pentium IV 1.8 GHz (1 Gb RAM) and are displayed in Table 4. We do not report the run times of NJ* and BIONJ*, as they are nearly the same as those of MVR*. In fact, NJ* and BIONJ* are ~2% faster than MVR*, because they are simpler, but these simplifications does not concern the heavy *O*(*n*^3^) parts of the algorithms (see Methods). We also report the run times of SDM [6], which are in the same range as the fastest tree building algorithms, except with Driskell et al. [20]-like datasets, where SDM has to summarize a large number (254) of source matrices, but where the number of taxa (69) is relatively low. In this case, the run time of SDM is analogous to that of FITCH and MW* and remains quite handy (~5 minutes per dataset).

**Table 4:** Run times

**(a): 25% taxon deletion rate**										
	**SDM**		**FITCH**			MW*			MVR*	

	*K *=		*k *=			*k *=			*k *=	
	10	2	10	20	2	10	20	2	10	20

*n *= 48	< 1	11	23	23	21	39	41	< 1	< 1	< 1
*n *= 96	5	437	482	479	623	932	926	7	6	5
*n *= 192	32	11,065	13,864	13,945	23,541	34,368	35,017	57	60	42

										

**(b): 75% taxon deletion rate**										

	**SDM**		**FITCH**			MW*			MVR*	

	*k *=		*k *=			*k *=			*k *=	
	10	2	10	20	2	10	20	2	10	20

*n *= 48	< 1	6	17	23	10	28	36	< 1	< 1	< 1
*n *= 96	< 1	22	455	492	29	656	667	< 1	4	7
*n *= 192	2	448	11,532	14,025	916	32,371	34,152	3	31	52

										

**(c): 1.2% missing distances (Driskell et al.)**										

	**SDM**		**FITCH**			MW*			MVR*	

	*k *= 254		*k *= 254			*k *= 254			*k *= 254	

*n *= 48	334		132			268			< 1	

Mean run times are provided for various taxon numbers (*n*), gene numbers (*k*) and proportions of missing entries: (a) 25%, (b) 75%, and (c) 1.2% using Driskell et al. [20]-like datasets. Run times are measured in seconds using a standard PC (Pentium IV 1.8 GHz, 1Gb RAM). The low run times with *k *= 2 and 75% deletion rate are explained by the low size of the distance super-matrices (see text for explanations).

As expected from their mere principle, the run times of the various tree building algorithms are not much affected by the proportion of missing distances, which is induced by the taxon deletion rate (25% or 75%) and the number of source matrices (*k*). The only apparent exceptions correspond to *k *= 2 and 75% deletion rate, where all algorithms seem to be quite fast; but in this case the distance supermatrices are of low size (~20, ~42 and ~85 for *n *equal to 48, 96 and 192, respectively), which explains this finding. Indeed, in this case it occurs frequently that some taxa have no gene (among 2) in common with any of the other taxa, and such taxa cannot be analyzed as all their distances to the other taxa are missing.

With 25% taxon deletion proportion, *n *= 48 and *k *= 10, run times of ~3 hours and ~5 hours are required by FITCH and MW*, respectively, to build the 500 trees corresponding to all gene collections in any given gene combination level. The same task, which induces calculations similar to bootstrapping, is achieved in ~30 seconds by any of our agglomerative algorithms. The difference between the agglomerative algorithms and the others increases when the number of taxa increases, as expected given that their time complexity are *O*(*sn*^3^) (i.e. *O*(*n*^3^) as *s *is kept constant) and *O*(*n*^4^) or more, respectively. With 192 taxa, FITCH and MW* require more than 3 hours to build a single tree, while the agglomerative algorithms require less than 1 minute; this run time makes easy to perform a bootstrap study with our algorithms, but pretty much impossible with FITCH or MW*. With even larger datasets (say, above 500 taxa) neither FITCH nor MW* can be used to build a single tree, while our algorithms still run in a few minutes.

## Conclusion

Thanks to the ever increasing flow of sequence data, phylogenomic analyses and supertree buildings are more and more frequently used to draw the evolutionary tree of living species. Larger and larger datasets are processed, requiring sophisticated approaches and algorithms. In this context, distance-based methods are quite useful, as they are both very fast and fairly accurate. New techniques, such as SDM [6], allow quickly estimating distance supermatrices that summarize the topological signal being contained in a collection of source distance matrices or gene trees. However, these supermatrices may be incomplete due to low taxon coverage in the selected genes. In this (common) case, fast distance-based tree building algorithms such as NJ, BIONJ, FASTME or STC are no longer applicable.

This paper presents an adaptation to incomplete distance matrices of several agglomerative algorithms, namely NJ, BIONJ and MVR. We show that the formulae forming the basis of these algorithms can be rewritten to account for missing distances. Moreover, the same holds for the quartet-based pair selection criterion of ADDTREE. Combining both NJ and ADDTREE generalized pair selection criteria, we obtain fast and accurate algorithms that require *O*(*n*^3^) run times, where *n *is the number of taxa, i.e. run times that are similar to NJ's. These three novel algorithms, named NJ*, BIONJ* and MVR*, show (in our simulations) topological accuracy similar or higher to that of FITCH and MW*, which are much more time consuming. MVR* appears to be best, followed by BIONJ*. In a phylogenomic context, MVR* accounts for (and benefits from, regarding other algorithms) the fact that gene distribution among species is very heterogeneous, which implies that some distances are accurately estimated (using numerous genes) while some others are poorly estimated (with few genes). Combined with the SDM method [6] to estimate distance supermatrices, MVR* and BIONJ* are relevant alternatives to standard supertree techniques [7], as MRP [51,52]. JAVA implementations of these algorithms are available in PhyD* software and downloadable from [8]. All our datasets are also available from this URL.

Several research directions would deserve to be explored. The variances and covariances of the distance estimates in the distance supermatrix could be accounted for in a more complete and accurate way, e.g. in the line of WEIGHBOR [40] for the pair selection criterion, or using the generalized least-squares version of MVR [4]. There is a clear need for a pair selection criterion being able to point out *xy *taxon pairs, even when the corresponding Δ_*xy *_distance is missing. Theoretical results highlighting the cases where our algorithms will succeed (or fail) in recovering the correct tree, would likely help to improve these algorithms or design new ones. Adapting to missing distances very fast algorithms [41-46] could be promising. Finally, dealing with missing distances is likely required in other (non phylogenomic) applications of phylogenetic trees, and in related problems, as phylogenetic network inference [60].

## Methods

Existing agglomerative algorithms are defined by criteria and formulae which all can be rewritten as distance averages. These algorithms (e.g. NJ [1,2], BIONJ [3] and MVR [4]) are generalized to incomplete distance matrices by estimating these averages using available distances, when some of those are missing. In the following, we first define notation and present a generic agglomerative scheme that covers all the algorithms being discussed here. Then, we describe for each of the three agglomeration steps (pair selection, branch length estimation, and matrix reduction), how NJ is generalized into NJ* to deal with missing distances. NJ* is further refined by BIONJ* that incorporates a first simple estimation of the variance associated to each evolutionary distance. Finally, a second, more accurate estimation of this variance is used by MVR* that generalizes the weighted least-squares (WLS) version of the MVR [4] approach.

### Notation

Let *L*_*n *_= {1,2, ..., *n*} be the set of all taxa numbered from 1 to *n*, and (Δ_*ij*_) a distance matrix, where Δ_*ij *_corresponds to the evolutionary distance between taxa *i*, *j *∈ *L*_*n*_, and Δ_*ii *_= 0, ∀*i *∈ *L*_*n*_. Distance-based algorithms build a tree *T *(also denoted as $\hat{T}$, depending on the context) from (Δ_*ij*_), and estimate all branch lengths *T*_*uv*_, where *uv *is any pair of sibling nodes in *T*. At each agglomeration stage, a taxon pair *xy *is selected, connected to a new internal node *u*, and replaced by *u *in (Δ_*ij*_). Thus, at each stage, the set *L*_*r *_= {1,2, ..., r} of non-agglomerated taxa drops in cardinality by 1, and *r *is changed into *r *- 1. Tree reconstruction stops when *r *= 2.

### Agglomerative algorithms with complete distance matrices

A number of existing agglomerative algorithms to deal with complete matrices can be summarized using the following scheme [4]:

• Input *L*_*n *_= {1,2, ..., *n*} and (Δ_*ij*_);

• *r *= *n*;

• While *r *> 2, do:

(a) Select the *xy *pair to be merged into *u *by optimizing an agglomeration criterion;

(b) Estimate the branch lengths *T*_*xu *_and *T*_*yu*_:

(1) $$T_{xu}=\Delta_{xy}-T_{yu}=\frac{1}{2}\Delta_{xy}+\sum_{i\in L_{r}-\{x,y\}}w_{i}\left(\Delta_{xi}-\Delta_{yi}\right)$$
					 with 
						$$\sum_{i\in L_{r}-\{x,y\}}w_{i}=\frac{1}{2}$$
					;

(c) Reduce the distance matrix (Δ_*ij*_) for all *i *≠ *x*, *y*:

(2) Δ_*ui *_= *λ*_*i *_(Δ_*xi *_- *T*_*xu*_) + (1 - *λ*_*i*_)(Δ_*yi *_- *T*_*yu*_) with *λ*_*i *_∈ [0,1]

(d) *r *= *r *- 1;

• Output *T*.

Step (a) in this generic scheme searches for the taxon pair *xy *to be merged by optimizing an agglomeration criterion. NJ, BIONJ and MVR select the pair which maximizes [1,2]:

(3) $$\begin{array}{ccc} Q_{xy}=R_{x}+R_{y}-\left(r-2\right)\Delta_{xy}, & \text{where} & R_{z}=\sum_{i\in L_{r}}\Delta_{zi}. \end{array}$$

Let (Δ_*ij*_) be additive [61], i.e. be defined as the path-length distance between taxa in a phylogenetic tree *T *with positive branch lengths; then, maximizing *Q*_*xy *_over all taxon pairs selects a cherry of *T*, i.e. a pair of taxa being separated by a unique internal node in *T*. In other words, *Q*_*xy *_is consistent [36]. However, it is easily shown (using counter-examples) that the second best taxon pair (based on *Q*_*xy *_values) is not necessarily a cherry of *T*.

Conversely, the ADDTREE [5] pair selection criterion implies that all cherries of *T *have highest criterion value. The ADDTREE criterion counts the number of times where the *xy *pair is a cherry in all taxon quartets *xyij*:

(4) $$N_{xy}=\sum_{i<j\in L_{r}-\{x,y\}}H\left(\Delta_{xi}+\Delta_{yj}-\Delta_{xy}-\Delta_{ij}\right)H\left(\Delta_{xj}+\Delta_{yi}-\Delta_{xy}-\Delta_{ij}\right)$$

where *H*(*t*) = 1 if t ≥ 0, and *H*(*t*) = 0 if *t *< 0. This criterion has integer values ranging from 0 to (*n *- 2)(*n *- 3)/2, and this maximum value is reached for all cherries (but for the cherries only) with additive distance matrices. Careful implementation [39] of ADDTREE allows for *O*(*n*^4^) run time. NJ, BIONJ and MVR are much faster. They first compute all *R*_*z *_sums in Equation (3), and then compute in *O*(1) the *Q*_*xy *_value of each *xy *pair. Each agglomeration stage thus requires *O*(*r*^2^) time (branch-length estimation and matrix reduction are achieved in *O*(*r*)), and the whole algorithm is in *O*(*n*^3^). Moreover, *Q*_*xy *_can be seen as a continuous version of *N*_*xy *_[62].

After *xy *pair selection, *x *and *y *are connected to the new node *u*, and the lengths of *xu *and *yu *branches are estimated using Equation (1). Assuming that (Δ_*ij*_) is additive and corresponds to tree *T*, we have *T*_*xu *_= (Δ_*xy *_+ Δ_*xi *_- Δ_*yi*_)/2, ∀*i *≠ *x*, *y*. Equation (1) averages these elementary estimators using various (*w*_*i*_) weightings. With NJ, the average is equally-weighted and we have *w*_*i *_= *w *= 1/(2(*r *- 2)). We shall see that MVR uses different *w*_*i *_weights.

Finally (step (c)), (Δ_*ij*_) is reduced by replacing *x *and *y *with the new node *u*, and by computing all Δ_*ui *_distances, ∀*i *≠ *x*, *y*. When (Δ_*ij*_) is additive and corresponds to tree *T*, we have Δ_*ui *_= Δ_*xi *_- *T*_*xu *_= Δ_*yi *_- *T*_*yu*_. Equation (2) averages these two elementary estimators. NJ uses equal weights (λ_*i *_= 1 - λ_*i *_= 1/2) while BIONJ and MVR adjust λ_*i *_in order to minimize the variance of Δ_*ui *_and to have reliable distance estimates during all agglomeration stages. For this purpose, BIONJ and MVR use (approximate) models for the variances and covariances of the distance estimates in (Δ_*ij*_).

### NJ*: generalizing NJ to incomplete distance matrices

When (Δ_*ij*_) is incomplete (missing entries are denoted as ∅), the criteria and equations above do not apply. We shall see in this section how they are generalized to define the NJ* algorithm, which keeps NJ's *O*(*n*^3^) time complexity and is nearly equivalent to NJ with complete matrices.

#### (a) Agglomeration criterion

Let ${Q^{}}_{xy}$ = *Q*_*xy*_/(*r *- 2). Maximizing ${Q^{}}_{xy}$ is the same as maximizing *Q*_*xy *_(Equation (3)), and we have:

${Q^{\prime }}_{xy}=\frac{R_{xy}}{r-2}-\Delta_{xy}$, where $R_{xy}=\sum_{i\in L_{r}}\left(\Delta_{xi}+\Delta_{yi}\right)$,

which can be rewritten as:

(5) $${Q^{\prime }}_{xy}=\frac{2}{r-2}\Delta_{xy}+\frac{1}{r-2}\sum_{i\in L_{r}-\{x,y\}}\left(\Delta_{xi}+\Delta_{yi}-\Delta_{xy}\right).$$

The sum in Equation (5) relates to terms representing how distant is the path joining *x *to *y *from other taxa *i *≠ *x*, *y *(Δ_*xi *_+ Δ_*yi *_- Δ_*xy*_ equals twice the distance between *u *and *i*), whereas the first term expresses the additional distance induced by Δ_*xy*_. It has been shown [63,64] that the relative weight of these two factors is unique, due to consistency requirement, and ${Q^{\prime }}_{xy}$ can be interpreted as the mean acentrality of the *xy *pair [35,36]. To extend this criterion to incomplete distance matrices, we estimate it using the set of taxa with non-missing distances: $S_{xy}^{*}$ = {*i *∈ *L*_*r *_: Δ_*xi*_, Δ_*yi *_≠ ∅}. Moreover, we assume Δ_*xy *_≠ ∅, and thus *x*, *y *∈ $S_{xy}^{*}$. The normalization factor is then equal to |$S_{xy}^{*}$| - 2 (instead of *r *- 2) and we obtain the following generalization of Equation (5):

$$Q_{xy}^{*}=\frac{2}{\left|S_{xy}^{*}\right|-2}\Delta_{xy}+\frac{1}{\left|S_{xy}^{*}\right|-2}\sum_{i\in S_{xy}^{*}-\{x,y\}}\left(\Delta_{xi}+\Delta_{yi}-\Delta_{xy}\right),$$

which applies to incomplete distance matrices, and is identical to ${Q^{}}_{xy}$ with complete ones. This equation further simplifies into:

(6) $$\begin{array}{ccc} Q_{xy}^{*}=\frac{R_{xy}^{*}}{\left|S_{xy}^{*}\right|-2}-\Delta_{xy}, & \text{where} & R_{xy}^{*}=\sum_{i\in S_{xy}^{*}}\left(\Delta_{xi}+\Delta_{yi}\right). \end{array}$$

Other solutions are possible to extend Equation (5), e.g. preserving Δ_*xy*_/(*r *- 2) term rather than transforming it into Δ_*xy*_/($S_{xy}^{*}$ - 2). Simulation results (not shown) indicate that criterion (6) has better topological accuracy than these alternatives. Theoretical results would be desirable to explain these observations and establish the properties of criterion (6), but a first simple explanation is that Equation (6) precisely corresponds to the ${Q^{}}_{xy}$ value being computed on $S_{xy}^{*}$ taxon subset. To be consistent on the whole set of taxa (*L*_*r*_), it is mandatory that the criterion is consistent on taxon subsets ($S_{xy}^{*}$, here), and Equation (6) satisfies this requirement.

Maximizing $Q_{xy}^{*}$ seems to require *O*(*r*^3^) time for each iteration, and thus a total time complexity of *O*(*n*^4^). However, efficient implementation allows for *O*(*n*^3^) total run time. At the first stage (*r *= *n*), $R_{xy}^{*}$ and |$S_{xy}^{*}$| values are computed and stored for all *x*, *y ∈ L*_*n*_, which requires *O*(*n*^3^) time. In the subsequent agglomeration stages, these values are updated as follows:

• After step (a), for all *i*, *j *∈ *L*_*r *_- {*x*, *y*} we remove from $R_{ij}^{*}$ and |$S_{ij}^{*}$| : Δ_*xi *_and Δ_*xj *_(if Δ_*xi *_≠ ∅ and Δ_*xj *_≠ ∅), and Δ_*yi *_and Δ_*yj *_(if Δ_*ui *_≠ ∅ and Δ_*uj *_≠ ∅).

• After step (c), we compute $R_{ui}^{*}$ and |$S_{ui}^{*}$| for all *i *∈ *L*_*r *_- {u}, and

• for all *i*, *j* ∈ *L*_*r *_- {u}, we add Δ_*ui *_and Δ_*uj *_to $R_{ij}^{*}$ and |$S_{ij}^{*}$| (if Δ_*ui *_≠ ∅ and Δ_*uj *_≠ ∅).

Each of these three updating routines requires *O*(*r*^2^) time, just as pair selection using criterion (6), meaning that using $Q_{xy}^{*}$ instead of *Q*_*xy *_does not change the total *O*(*n*^3^) time complexity of the original NJ algorithm.

However, as discussed earlier, a limitation of criterion $Q_{xy}^{*}$ is that: (1) it cannot be computed when Δ_*xy *_= ∅, and (2) only the best pair is guaranteed (with additive distance) to be a cherry in the correct tree. When *xy *is the best pair in the complete additive distance matrix, but Δ_*xy *_is missing in the available distance matrix, then using $Q_{xy}^{*}$ does not provide any guaranty of correctness. This difficulty is partly alleviated when using a generalization of *N*_*xy*_, as this criterion selects all cherries in the correct tree with complete additive distances. When some of the cherries correspond to missing distances, we are still able to select the others that correspond to non-missing entries. Our generalization of *N*_*xy *_(Equation (4)) to incomplete distances is defined as follows. Let:

(7) $${\tilde{N}}_{xy}^{*}=\sum_{i,j\in C_{xy}^{*}}H\left(\Delta_{xi}+\Delta_{yj}-\Delta_{xy}-\Delta_{ij}\right),$$

(8) $$\text{where }C_{xy}^{*}=\left\{(i,j)\neq (x,y),(y,x):i\neq j,\Delta_{xi},\Delta_{yj},\Delta_{ij}\neq \emptyset \right\}.$$

${\tilde{N}}_{xy}^{*}$ differs from *N*_*xy *_in that we sum both *H *terms, instead of multiplying them. This way we exploit all available information. Indeed, when Δ_*xj *_= ∅ and/or Δ_*yi *_= ∅ but the other entries are available, we still use *H*(Δ_*xi *_+ Δ_*yi *_- Δ_*xy *_- Δ_*ij*_) in ${\tilde{N}}_{xy}^{*}$ while a multiplicative solution in the line of *N*_*xy *_would discard this term. Moreover, it is easily seen that ${\tilde{N}}_{xy}^{*}$ = 2*N*_*xy *_with complete additive distances. To select among taxon pairs, we use the averaged form of ${\tilde{N}}_{xy}^{*}$, that is:

(9) $$N_{xy}^{*}=\frac{{\tilde{N}}_{xy}^{*}}{\left|C_{xy}^{*}\right|},$$

which expresses the mean number of quartets where the *xy *pair corresponds to a cherry.

However, selecting pairs using $N_{xy}^{*}$ sometimes produces ties. In this case, we select the pair with higher |$C_{xy}^{*}$| value, that is the pair which is supported by the larger number of quartets. But ties may still occur, in which case we use:

(10) $$M_{xy}^{*}=\left|Miss(x)-Miss(y)\right|+\left|Miss(y)-Mass(x)\right|,$$

where *Miss*(*z*) = {*i *∈ *L*_*r*_, ≠ *z *: Δ_*iz *_= ∅ } corresponds to missing entries for taxon *z*. $M_{xy}^{*}$ counts the number of missing entries in the current matrix that will be removed in the next step (see reduction procedure (13)). Maximizing $M_{xy}^{*}$ tends to quickly fill missing entries in the running distance matrix, which both frees from Δ_*xy *_≠ ∅ limitation and allows using *Q*_*xy *_pair selection criterion only. Finally, in some (very rare) cases, we still have ties and then maximize the continuous version [62] of ${\tilde{N}}_{xy}^{*}$:

(11) $${N^{\prime }}_{xy}^{*}=\sum_{(i,j)\in C_{xy}^{*}}\left(\Delta_{xi}+\Delta_{yj}-\Delta_{xy}-\Delta_{ij}\right).$$

Pair selection criteria $N_{xy}^{*}$ (9), |$C_{xy}^{*}$| (8), $M_{xy}^{*}$ (10) and ${N^{\prime }}_{xy}^{*}$ (11) are used in a lexicographic way: taxon pairs are ranked based on the first criterion (${\overset{}{N}}_{xy}^{*}$), the second one (|$C_{xy}^{*}$|) is used in case of ties, etc. However, using these four criteria only would result in *O*(*n*^4^) time complexity. In order to preserve *O*(*n*^3^) run times, we first select the *s *top pairs based on $Q_{xy}^{*}$ criterion (6), and then use the other criteria in lexicographic order to select the pair to be agglomerated among these *s *pairs. As computing Equations (7) to (11) requires *O*(*r*^2^) or less per taxon pair, the total time complexity of pair selection is *O*(*n*^3^) (first selection using (6)) plus *O*(*s*∑*r*^2^) (final selection using (8) to (11)), i.e. *O*(*n*^3^). As explained above, $Q_{xy}^{*}$ does not provide any guaranty of correctness with missing distances, while $N_{xy}^{*}$ and ${N^{\prime }}_{xy}^{*}$ partly circumvent the difficulty. However, $Q_{xy}^{*}$ enables to extract the most promising pairs for agglomeration and we have seen (Figure 1) that using for *s *a small constant (typically 15) is sufficient to obtain high accuracy, meaning that, in practice, run times are in *O*(*n*^3^).

#### (b) Branch length estimation

Equation (1) is easily rewritten using non-missing entries only:

(12) $$\begin{array}{ccc} T_{xu}=\Delta_{xy}-T_{yu}=\frac{1}{2}\Delta_{xy}+\sum_{i\in S_{xy}^{*}-\{x,y\}}w_{i}\left(\Delta_{xi}-\Delta_{yi}\right), & \text{where} & \sum_{i\in S_{xy}^{*}-\{x,y\}}w_{i}=1/2. \end{array}$$

NJ uses the same weight *w*_*i *_for every taxon *i*. The same holds for NJ*, that is, *w*_*i *_= *w *= 1/(2(|$S_{xy}^{*}$| - 2)). Note that for the selected pair we have Δ_*xy*_, $S_{xy}^{*}$ ≠ ∅, meaning that Equation (12) is always applicable. Just as with NJ, branch length estimation (12) requires *O*(*r*) time at each agglomeration stage.

#### (c) Matrix reduction

Equation (2) averages two elementary estimators, and with NJ this average is equally weighted. With missing distances it may occur that one of these two estimators is not applicable (e.g. when Δ_*xi *_≠ ∅), that both are applicable, or that none is applicable. Thus, in NJ* Equation (2) becomes:

(13) $$\Delta_{ui}=\{\begin{array}{lll} \lambda_{i}(\Delta_{xi}-T_{xu})+(1-\lambda_{i})(\Delta_{yi}-T_{yu}) & \text{when} & \Delta_{xi}\neq \emptyset \text{ and }\Delta_{yi}\neq \emptyset ,\\ \Delta_{xi}-T_{xu} & \text{when} & \Delta_{xi}\neq \emptyset \;\text{and }\Delta_{yi}=\emptyset ,\\ \Delta_{yi}-T_{yu} & \text{when} & \Delta_{xi}=\emptyset \;\text{and }\Delta_{yi}\neq \emptyset ,\\ \emptyset & \text{when} & \Delta_{xi}=\Delta_{yi}=\emptyset , \end{array}$$

where *λ*_*i *_= *λ *= 1/2. In the second and third cases, entries missing in the previous matrix are now present in the new, reduced matrix. We have seen that criterion (10) tends to maximize the number of such entries, in order to fill as fast as possible the missing distances in the running matrix. Just as branch length estimation (12), matrix reduction (13) requires *O*(*r*) time at each stage and does not impact total time complexity. Thus, NJ* requires *O*(*n*^3^) run times, when *s *is kept constant.

### BIONJ*: improving the reduction step, a first simple solution

BIONJ* uses the same pair selection criteria as NJ*, and adapts to missing distances BIONJ reduction procedure. BIONJ uses the degree of freedom corresponding to the *λ*_*i *_parameter in Equation (2), in order to minimize the variance of the new Δ_*ui *_estimates in step (c). For this purpose, BIONJ assumes a simple Poisson model of the variances in the original (Δ_*ij*_) matrix, stating that the variance *V*_*ij *_of Δ_*ij *_is proportional to Δ_*ij*_. BIONJ also accounts for the covariances in (Δ_*ij*_) (see [3] for more details). It uses a single λ parameter for every *xy *pair, which does not depend on *i *and is given by

(14) $$\lambda_{i}=\lambda =\frac{1}{2}+\frac{1}{2(r-2)V_{xy}}\sum_{j\in L_{r}-\{x,y\}}\left(V_{yj}-V_{xj}\right).$$

Again, this equation may be seen as an average and can be rewritten using available entries only as:

(15) $$\lambda_{i}^{*}=\lambda^{*}=\frac{1}{2}+\frac{1}{2\left(\left|S_{xy}^{*}\right|-2\right)V_{xy}}\sum_{j\in S_{xy}^{*}-\{x,y\}}\left(V_{yj}-V_{xj}\right).$$

The reduction step (c) is achieved by BIONJ* as defined by Equation (13), but using so-defined λ* (instead of 1/2) when Δ_*xi *_≠ ∅ and Δ_*yi *_≠ ∅.

Moreover, BIONJ starts with variance matrix (*V*_*ij*_) = (Δ_*ij*_) and reduces this matrix at each stage using *λ *value from Equation (14) and equation:

*V*_*ui *_= *λV*_*xi *_+ (1-*λ*)*V*_*yi *_- *λ*(1 - *λ*)*V*_*xy*_.

BIONJ* combines this formula with Equation (13) and (15) to reduce the variance matrix, that is:

(16) $$V_{ui}=\{\begin{array}{lll} \lambda^{*}V_{xi}+(1-\lambda^{*})V_{yi}-\lambda^{*}(1-\lambda^{*})V_{xy} & \text{when} & \Delta_{xi}\neq \emptyset \text{ and }\Delta_{yi}\neq \emptyset ,\\ V_{xi} & \text{when} & \Delta_{xi}\neq \emptyset \;\text{and }\Delta_{yi}=\emptyset ,\\ V_{yi} & \text{when} & \Delta_{xi}=\emptyset \;\text{and }\Delta_{yi}\neq \emptyset ,\\ \emptyset & \text{when} & \Delta_{xi}=\Delta_{yi}=\emptyset , \end{array}$$

Computing λ* using Equation (15) and achieving matrix reductions (13) and (16) requires *O*(*r*) run times. Thus, BIONJ* has *O*(*n*^3^) time complexity (when *s *is kept constant, else *O*(*sn*^3^)).

### MVR*: improving BIONJ* using variances dedicated to distance supermatrices

The BIONJ variance model is well suited for one-gene studies where distance estimations all use the same number of sites (at least when gaps are removed). With phylogenomic studies, some distances are computed using a large number of genes, and thus are reliable, while other distances are based on a few genes and are poorly estimated. Moreover, some distances may be missing due to the absence of common genes between the two species being compared. Altogether, this implies that the BIONJ and BIONJ* variance model can be improved to better fit phylogenomic requirements. This section describes the MVR* algorithm that is intended to this purpose.

Steps (b) and (c) in the generic scheme are based on *w*_*i *_and λ_*i *_parameters, respectively. The MVR algorithm [4] generalizes the BIONJ approach and uses these degrees of freedom in order to minimize the variance of the new estimates *T*_*ux*_, *T*_*uy *_and Δ_*ui*_. The main difference from BIONJ is that MVR is able to deal with any variance-covariance model of the δ_*ij *_distance estimates, while BIONJ is restricted to the Poisson model. The MVR variant that we use here only considers the variances and neglects the covariances, thus assuming a weighted least-squares model (it was called MVR-WLS in [4], but is named MVR here for simplicity). Thus, MVR inputs a distance matrix (Δ_*ij*_) and the corresponding (*V*_*ij*_) variance matrix. We shall see in the next section how (*V*_*ij*_) is calculated to deal with phylogenomic data, and describe now the way MVR and MVR* use and update these matrices all along the agglomeration procedure.

MVR uses *Q*_*xy *_pair selection criterion (3), just as NJ and BIONJ, while MVR* uses the same criteria and selection procedure as NJ* and BIONJ*.

In MVR step (b), i.e. branch length estimation, *w*_*i *_weights in Equation (1) depend on *i *and are given by:

(17) $$\begin{array}{l} w_{i}=\frac{\mu }{V_{xi}+V_{yi}},\\ \text{with normalization term }\mu =\frac{1}{2}{\left(\sum_{i\in L_{r}-\{x,y\}}\frac{1}{V_{xi}+V_{yi}}\right)}^{-1}. \end{array}$$

MVR* uses Equation (12) (instead of Equation (1)) to deal with missing entries, and adapts above Equation (17) by replacing *L*_*r *_by $S_{xy}^{*}$.

In MVR step (c), i.e. matrix reduction, a different *λ*_*i *_parameter is associated in Equation (2) to each taxon *i *≠ *x*, *y *using:

(18) $$\lambda_{i}=\frac{V_{yi}}{V_{xi}+V_{yi}}.$$

This value puts more weight and confidence on (Δ_*xi *_- *T*_*xu*_) when the associated variance *V*_*xi *_is low, compared to *V*_*yi*_. Equation (18) is also used by MVR*, but combined with Equation (13) to deal with missing distances.

Finally, MVR (just like BIONJ) reduces the variance matrix at each agglomeration stage. To this purpose, MVR uses the following equation:

$$V_{ui}=\frac{V_{xi}V_{yi}}{V_{xi}+V_{yi}}.$$

This equation is also used by MVR* in combination with Equation (16).

All the computations described above (except pair selection) require *O*(*r*) run times at each agglomeration stage, and thus MVR* has *O*(*n*^3^) time complexity, just as do NJ* and BIONJ*.

### Estimating the variances associated to distance supermatrices

Distance supermatrices are computed [6,19] from source matrices which are first rescaled, and then averaged. SDM [6] inputs a collection *C *= $\left\{\left(\Delta_{ij}^{1}\right),\left(\Delta_{ij}^{2}\right),...,\left(\Delta_{ij}^{k}\right)\right\}$ of *k *distance matrices – each defined on taxon set *L*_*p *_and estimated from sequences with size *s*_*p*_—, and deforms them, without changing their topological signal, so as to bring them as close as possible to each other before averaging. The first deformation is scaling, which multiplies each ($\Delta_{ij}^{p}$) distance matrix by a factor α_*p*_. The second (optional in SDM) deformation adds a constant a_*ip *_to every non-diagonal $\Delta_{ij}^{p}$ entries. Then, SDM averages the resulting modified matrices to obtain the ($\Delta_{ij}^{\text{SDM}}$) super-matrix that is defined by:

(19) $$\begin{array}{ccc} \Delta_{ij}^{\text{SDM}}=\frac{1}{W_{ij}}\sum_{1\leq p\leq k,L_{p}⊃\{i,j\}}s_{p}\left(\alpha_{p}\Delta_{ij}^{p}+a_{ip}+a_{jp}\right), & \text{where} & W_{ij}=\sum_{1\leq p\leq k,L_{p}⊃\{i,j\}}s_{p}. \end{array}$$

Neglecting the variance of the deformation factors, we obtain a simple expression of the variance of $\Delta_{ij}^{\text{SDM}}$:

(20) $$V_{ij}^{\text{SDM}}=\frac{1}{W_{ij}^{2}}\sum_{1\leq p\leq k,L_{p}⊃\{i,j\}}s_{p}^{2}\alpha_{p}^{2}V_{ij}^{p},$$

where $V_{ij}^{p}$ is the variance of $\Delta_{ij}^{p}$. Note that no covariance terms between any $\Delta_{ij}^{p}$ and $\Delta_{ij}^{q}$ estimates appear in Equation (20), as these source distances are estimated from different genes and are independent. Moreover, the covariances between the entries in the SDM supermatrix are neglected, as is the case in a number of (WLS) approaches [30,32,40].

Several studies have shown that the variance *V*_*ij *_associated with the evolutionary distance Δ_*ij *_(estimated from a single gene) is approximately equal to $\Delta_{ij}^{\rho }/s$ with ρ ≈ 2 [11,65]. Based on various experiments (not shown), we have chosen the usual formula $V_{ij}=\Delta_{ij}^{2}/s$, which corresponds to default option in FITCH program. Equation (20) then becomes:

$$V_{ij}^{\text{SDM}}=\frac{1}{W_{ij}^{2}}\sum_{1\leq p\leq k,L_{p}⊃\{i,j\}}s_{p}{\left(\alpha_{p}\Delta_{ij}^{p}\right)}^{2}.$$

## Authors' contributions

AC designed and implemented the algorithms and experiments, performed the computations that are shown here, and wrote the manuscript. OG supervised these works, participated in the design of algorithms and experiments, and wrote the manuscript.
